# Fatty acid synthase inhibitor cerulenin attenuates glioblastoma progression by reducing EMT and stemness phenotypes, inducing oxidative and ER stress response, and targeting PI3K/AKT/NF-κB axis

**DOI:** 10.1007/s12032-025-02697-2

**Published:** 2025-03-25

**Authors:** Murat Pekmez, Şefika Beyza Mete, Yunus Aksüt, İrem Öğütcü, Fatma Nur Baştürk, Yusuf Can Gerçek, Aslıhan Şengelen

**Affiliations:** 1 Department of Molecular Biology and Genetics, Faculty of Science, Istanbul University, Balabanağa, Şehzadebaşı RoadVezneciler-Fatih, 34134 Istanbul, Türkiye; 2 Department of Molecular Biology and Genetics, Institute of Graduate Studies in Sciences, Istanbul University, Istanbul, Türkiye; 3 Department of Molecular Biology and Genetics, Basic Medical Sciences, School of Medicine, Koç University, Istanbul, Türkiye; 4 Department of Biology, Institute of Graduate Studies in Sciences, Istanbul University, Istanbul, Türkiye; 5 Department of Biology, Faculty of Science, Istanbul University, Istanbul, Türkiye; 6 Centre for Plant and Herbal Products Research-Development, Istanbul, Türkiye

**Keywords:** Glioblastoma, Cerulenin (CER), Fatty acid synthase (FASN) inhibitor, Cellular stress response, Anti-cancer effect

## Abstract

Targeting cellular metabolism is becoming a critical approach for stopping cancer progression. Limited information is available regarding the effects of inhibiting the lipogenic enzyme fatty acid synthase (FASN) in glioblastoma (GB) cells (grade-IV-astrocytoma), which have high invasion and low response to standard treatments. Herein, we used cerulenin (CER) to inhibit FASN. CER treatments (3.6 μg/mL/48 h and 5.55 μg/mL/48 h indicate IC_20_ and IC_50_ values, respectively) led to a dose- and time-dependent decrease in the viability of the U-87MG human GB cells. A significant decrease was detected in the levels of fatty acids, including palmitic acid, determined by GS-MS analysis. FASN inhibition attenuated cell motility, 2D and 3D-clonogenic survival, and cell differentiation characteristics (related markers of epithelial-mesenchymal transition/EMT and stemness). Moreover, treatments caused mitochondrial membrane potential (MMP) collapse and increased intracellular reactive oxygen species (ROS) levels. Protein aggregates and ER stress in the cells also increased. Remarkably, despite increased Hsp70 and p-HSF1 levels against induced cellular stress, CER promoted markedly autophagy and apoptosis. The network pharmacology approach revealed that protein and lipid kinases are crucial targets in cell signaling, and PI3K, AKT, and NF-κB levels were confirmed by immunoblotting. The results demonstrated for the first time that inhibiting FA production and FASN function induces cell death through ROS generation and ER stress while simultaneously reducing the motility and aggressiveness of U-87MG human glioblastoma cells by attenuating EMT and stemness phenotypes. Therefore, blocking lipid metabolism using CER may be considered as a good candidate for GB therapeutic option.

## Introduction

Glioblastoma (GB, grade-IV astrocytoma) is the most aggressive and lethal primary brain tumor due to its high invasion and low response to standard treatment methods. The average survival of GB patients is less than one year, and their prognosis is still poor [1]. Focusing on the metabolic activity of GB cells, which proliferate rapidly and have a higher demand for biomolecules than healthy cells, is a promising research area. In particular, targeting/reducing the levels/synthases of fatty acids (FA, lipid-mediators) may be a good strategy to control glioma growth and spread [2]. Because, increased FA production helps to form cell membrane components and provides the energy demand of rapidly proliferating cells [3]. Gliomas have an accelerated de novo lipogenesis flux and often display the upregulation of FA synthase (FASN). This key metabolic multi-enzyme catalyzes reactions to produce palmitate (a fatty acid with a 16-carbon chain) [4]. FASN-related lipogenic phenotype is related to malignancy, stemness, and therapy resistance and is also a hallmark of invasive cancers. Notably, FASN was described to be overexpressed in glioma tissue and glioma-derived extracellular vesicles [5, 6]. Increasing reports showed that inhibiting the lipogenic enzyme FASN can be a potential therapeutic target for stopping glioma progression [7]. A FASN inhibitor, cerulenin (CER), reduced stemness marker expression in patient-derived glioma stem cells [8]. Blocking of FASN with specific shRNA or inhibitor-C75 was reported to suppress neovascularization by regulating the expression of VEGF-A in gliomas [9].

Impairment in lipid synthesis induces stress responses and cell death in cancer cells [3]. A study demonstrated that treating glioma cells with CER decreased endogenous FA synthesis by ~ 50% and induced S-phase cell arrest and apoptosis [6]. FASN inhibition was shown to cause degradation of mitochondrial membrane potential (MtMP) and an exaggerated increase in reactive oxygen species (ROS) that promotes apoptosis [10]. Besides, Chen et al. [11] revealed that the inhibition of protein palmitoylation, crucial for GB cell survival, with 2-bromopalmitate, tunicamycin, and CER resulted in G2-phase cell arrest and apoptosis through enhanced endoplasmic reticulum (ER) stress. Although it is well-known that FASN inhibition induces ER stress [12], there is insufficient information regarding its effect on stress proteins (heat shock proteins, Hsp) that are closely associated with unfolded protein response (UPR) [13], treatment resistance, motility, and stem cell behavior [14]. A few studies showed that the inhibitor CER increases Hsp70 in prostate cancer cells [15] and chicken muscle [16]. Moreover, FASN inhibitor treatments in various cancers also reduced cell migration and epithelial-mesenchymal transition (EMT) [17], which are crucial for the aggressiveness and cancer stem cell (CSC) properties of tumor cells [18]. However, the impact of FASN inhibition on the EMT of glioma cells is unknown. Therefore, herein, we used the most well-known lipid synthesis blocking agent CER, a natural antimycotic found in the fungus *Cephalosporium caerulens*, to inhibit FASN [19]. We aimed to investigate the effects of CER on GB cell survival, signaling mechanisms, migration, colony-forming and cellular stress management abilities, EMT and stem cell behaviors, oxidative damage, ER stress, and apoptotic responses. We also explored potential target molecules by revealing the network pharmacology of CER and GB.

The constraints and challenges associated with traditional treatments for GB have prompted the exploration of novel therapeutic approaches. Understanding the metabolic basis of GB and finding new treatments is an important objective. Utilizing CER, a significant FASN inhibitor, to study FA metabolism—one of the key metabolic pathways for GB—is a useful strategy for acquiring information. The current study’s results indicated that treatments with the FASN inhibitor CER decrease the viability of glioma cells in a manner dependent on both dosage and duration. A significant decrease was detected in the levels of fatty acids, including palmitic acid. Inhibitor therapy attenuated glioma cell motility and 2D and 3D-clonogenic survival, increased ROS production, led to MtMP loss, ER stress, and autophagy induction. Remarkably, despite increased Hsp70 and p-HSF1 levels, the treatment promoted cell death. Moreover, the network pharmacology approach revealed that protein and lipid kinases in cell signaling transduction are crucial targets, and phosphatidylinositol-mediated signaling [phosphatidylinositol 3-kinase (PI3K)/protein kinase B (PKB/AKT)/nuclear factor kappa B (NF-κB)] comes to the fore, as confirmed by immunoblotting. Overall, the results indicated for the first time that blocking FA production and FASN function provides cell death induction by ROS generation and ER stress induction and reduces the motility and aggressiveness of U-87MG human GB cells by attenuating EMT and stemness phenotypes. These observations demonstrate the importance of targeting lipid metabolism to overcome GB progression. Blocking metabolic activity using CER will also help establish novel combination therapy approaches by sensitizing the cell to chemotherapy.

## Materials and methods

### Cell culture and conditions

Human U-87MG and LN-229 glioblastoma cells and human embryonic kidney cells HEK-293 were obtained from Istanbul University Cell-Culture Collections and cultured in DMEM supplemented with 10% FBS, 1% penicillin/streptomycin/amphotericin-B solution, and 1% NEAA in a humidified atmosphere containing 5% CO_2_ at 37 °C. Main experiments were conducted on U-87MG at passages 3–10. Standard tissue culture reagents were obtained from Gibco (Carlsbad/USA).

### Cytotoxicity assay and cerulenin treatments

Cerulenin (CER, #C2389, ≥ 98% purity) was purchased from Sigma-Aldrich (St.Louis/USA) and dissolved in dimethyl sulfoxide (DMSO; Merck, Darmstadt/Germany). To determine the effects of CER on U-87MG cell viability and its IC_50_-value, MTT assay (NeoFroxx, Einhausen/Germany) was performed[20]. Briefly, exponentially growing-cells were plated into 96-well microplates (1 × 10^4^ cells/well), the following day, treated with CER in the range of 0–15 μg/mL for 24 h, 48 h, and 72 h. Next, MTT solution (5 mg/mL in D-PBS) was added for 4 h, followed by DMSO to dissolve the formazan crystals. The absorbance at 540 nm was measured. According to the sigmoidal dose–response curve, CER treatment doses were selected. Doses of 3.6 μg/mL (IC_20_-value = 80% cell viability) and 5.55 μg/mL (IC_50_-value = 50% cell viability) of CER were used for further analysis. Cells were seeded wells at specific densities (2 × 10^4^ in 8-well slides; 1 × 10^4^, 6 × 10^4^, and 3 × 10^5^ in 96-, 24-, and 6-well plates, respectively) and treated with CER for 48 h. DMSO final concentration in culture medium did not exceed 0.06% (v/v), and it was applied as the vehicle in the control group.

### Morphological cell changes

To evaluate the effect of CER treatments on the glioma cell morphology, untreated and treated U-87MG cells were photographed using a digital-camera attached (ToupTek Photonics XCAM-1080PHD, Zhejiang/China), inverted light microscope (Olympus/CKX31, Tokyo/Japan).

### Fatty acid extraction and profile determination by GC–MS analysis

To evaluate the effect of CER treatments on fatty acid composition, untreated and treated U-87MG cells in 6-well plates were harvested. The total lipid content of samples was determined using the Bligh and Dyer method [21]. Briefly, total lipids in each cell pellets (1 g) were extracted with 6 mL methanol:chloroform (2:1). Following the addition of chloroform (2 mL) to the mixture, thorough shaking was performed, dH_2_O (3.6 mL) was added, and samples were centrifuged (2000 rpm/10 min). The lower phase was extracted again with methanol (400µL) and chloroform (3.6 mL). The subphases were combined and the solvent was evaporated under nitrogen. The total lipid content (100 mg) was dissolved in n-Hexane (10 mL). Subsequently, KOH (2N) solution in methanol was added (100µL), vortexed (30 s), and samples were centrifuged (2000 rpm/10 min). The supernatant (1 mL) was transferred to a tube for gas chromatography-mass spectrometry (GC–MS) analyses [22]. Finally, methyl-esterified fatty acids were analyzed using an Agilent-7890AGC gas chromatograph (AgilentTechnologies, SantaClara/USA) and a 5975C MSD mass spectrometer (AgilentTechnologies). HP-88 (100mx0.25 mm-ID, 0.25 μm) column was used for separation. Helium served as carrier gas. The temperature program began at 120 °C/1 min, was increased at 5.5 °C/min to 175 °C for 10 min, then again increased at 7 °C/min to 210 °C for 5 min. The oven temperature was then raised to 240 °C in 5 °C increments over 3 min. Sample (1µL) from each vial was injected into column with a 1/20 split ratio. The mixed standard consisting of 37-component FAME (#CRM47885, Sigma) was used and sample peaks were identified according to the retention times of the standard substances.

### 2D- and 3D-colony formation assays

Colony-forming abilities of glioma cells were evaluated using both 2D- and 3D-culture systems. Post-treatment, U-87MG cells were collected and subsequently suspended in medium. For the 2D-colony formation assay (CFA), cells were plated into 6-well plates at a density of 1000 cells per well and incubated at 37 °C for 7 days. The cells were fixed in a 3:1 methanol:acetic acid mixture (5 min/37 °C), stained with 0.5% crystal-violet (15 min; Merck, Darmstadt/Germany), and then washed with distilled water. Plates were then air-dried and photographed. To conduct the 3D-soft agar colony formation assay (SA-CFA), a bottom layer with a culture medium containing 0.5% agar was prepared. Treated cells were suspended, mixed with 0.3% agar (final concentration), seeded coated 6-well plates (5000 cells/well), and incubated at 37 °C for 21 days, with fresh medium added every 3–4 days. Nitrotetrazolium blue chloride (NBT, Sigma, 1 mg/mL in D-PBS) was utilized for staining colonies at an incubation period of overnight at 37 °C. Cells/colonies were imaged using ChemiDoc-XRS/ImageLab-6.0.1-software (Bio-Rad, Hercules/USA) and counted using ImageJ-software.

### Migration and invasion assays

To assess CER treatment on cell migration, in vitro scratch assay was first performed. Following the initial 24 h-period of cell seeding in 24-well plates, a pipette tip was utilized to create the scratch area. The cell debris was removed by washing with D-PBS, followed by applying CER treatment. Images of untreated and treated U-87MG cells were taken at 0 h, and 48 h using an inverted light microscope (Olympus/CKX31) and analyzed using ImageJ software. Besides, transwell inserts with 8 μm-pore (Sarstedt/ Nümbrect-Germany), precoated with or without matrigel (50µL, #354,230, Corning/Wiesbaden-Germany), were utilized to assess the quantity of invited and migrated cells, respectively. Cells (3 × 10^4^ per well) were seeded in the upper chamber without FBS, while the lower chamber had medium with 10% FBS. Following 24 h at 37 °C, cells were fixed in 4% paraformaldehyde, stained with 0.5% crystal-violet, photographed using an inverted light microscope, and analyzed with ImageJ software.

### Double staining (Hoechst-33342/propidium iodide) apoptosis assay

To assess the impact of CER treatments on glioma cell apoptosis, U-87MG cells in chambered slides (8-well, NuncLab-TekII, Thermo/Invitrogen, Carlsbad/USA) were incubated with Hoechst-33342 (HO; #H-1399, Thermo/Invitrogen, blue-fluorescent dye for DNA staining) and propidium iodide (PI; #P1304MP, Thermo/Invitrogen, red-fluorescent dye for staining DNA of cells with compromised membrane integrity). Cells were stained with HO/PI (5 μg/mL in HBSS, 30 min/37 °C), washed with D-PBS, and examined by confocal fluorescence microscopy (Leica-SPE2, Germany). Excitation/emission wavelengths of 350/461 nm and 535/617 nm were used for HO and PI, respectively. The total cell count was determined using ImageJ-software. Apoptotic and dead cells were counted manually.

### Mitochondrial membrane potential assay

To assess the effect of CER treatments on glioma cells’ MtMP, U-87MG cells in chambered slides were incubated with JC-1 dye (1 μg/mL, 20 min/37 °C) according to the MtMP-kit protocol (#E-CK-A301, Elabscience, Beijing/China). Slides were then washed with D-PBS and examined by fluorescence microscopy (Olympus BX53 with DP73 digital-camera, Tokyo, Japan).

### Intracellular reactive oxygen species analysis

To measure intracellular ROS production, 2,7-dichlorofluorescein diacetate (H2DCF-DA), a cell-permeable, non-fluorescent probe (becomes highly fluorescent 2′,7′-DCF in the presence of ROS) was used. Following CER treatments, U-87MG cells in 96-well black culture plates were incubated with DCFH-DA (Sigma; 10 μM in HBBS, 30 min/37 °C). The fluorescence intensity was recorded over 2 h at 37 °C using a spectrofluorometer (FLx800, BioTek, Winooski/USA). Excitation/emission wavelength was 495/525 nm. Cell counting data was utilized to normalize the results.

#### Thioflavin T staining and ER stress quantification

To detect protein aggregation and, thus, ER stress after CER treatments, fluorescence-based imaging utilizing Thioflavin T (ThT; #T3516, Sigma) dye was conducted. Untreated and treated U-87MG cells in chambered slides were washed with D-PBS, fixated with 4% paraformaldehyde (PFA) solution (10 min/37 °C), and incubated with 50 mM ammonium chloride (NH_4_Cl) solution (10 min/37 °C) for quenching. Cells were permeabilized with 0.1% Triton X-100 in D-PBS (8 min/37 °C), then incubated with 20 μM ThT and 5 μg/mL Hoechst dye (30 min/37 °C), followed by a wash with D-PBS. Slides were examined under a confocal fluorescence microscope (Leica-SPE2) with 350/461 nm and 450/482 nm wavelengths for HO and ThT, respectively. ThT staining was analyzed using ImageJ-software.

### Protein isolation and Western blot analysis

Western blotting followed the standard procedure [20] to assess CER treatment effects on the target proteins. After harvesting untreated and treated U-87MG cells, total protein isolation was performed using ice-cold RIPA lysis and extraction buffer (#89,900, Thermo, Kwartsweg/Holland) with EDTA-free-PIC (Roche, Darmstadt/Germany). Protein concentration was determined utilizing the SMART™ BCA kit (iNtRON-Biotechnology, Seongnam/Korea). Proteins (30 μg/well) were separated by SDS-PAGE and transferred to a PVDF membrane (Thermo, Kwartsweg/Holland), then probed with primary and secondary antibodies (Table 1). After removing excess antibody, blots were visualized using ECL (SuperSignal West-Pico, Thermo, Kwartsweg/Holland) and ChemiDoc-XRS/ImageLab-6.0.1-software (Bio-Rad, Hercules/USA).

**Table 1:** Antibodies used for Western blotting

Antibody	Host	Dilution	Catalog Number	Company*
Anti-Bax	Rabbit	1:1000	50,599–2-Ig	Proteintech
Anti-Bcl-2	Rabbit	1:1000	12,789–1-AP	Proteintech
Anti-Cas-3/p17/p19	Rabbit	1:1000	19,677–1-AP	Proteintech
Anti-PARP1	Rabbit	1:1000	13,371–1-AP	Proteintech
Anti-LC3AI/II	Mouse	1:1000	PA5-22,990	Thermo/Invitrogen
Anti-Hsp60	Rabbit	1:1000	15,282–1-AP	Proteintech
Anti-Hsp70	Mouse	1:1000	MA3-007	Thermo/Invitrogen
Anti-Hsp90	Rabbit	1:1000	MA1-10,372	Thermo/Invitrogen
Anti-HSF1/p-HSF1^Ser303^	Rabbit	1:1000	PA5-114,582	Thermo/Invitrogen
Anti-Grp78	Rabbit	1:1000	NBP2-16,749	Novus
Anti-IRE1α	Rabbit	1:1000	NB100-2324	Novus
Anti-PERK	Mouse	1:1000	NBP1-51,661	Novus
Anti-ATF6	Mouse	1:1000	NBP1-40,256	Novus
Anti-TMED4	Rabbit	1:500	NB100-56393	Novus
Anti-PI3K	Rabbit	1:1000	MA1-74,183	Thermo/Invitrogen
Anti-p-PI3K^Tyr467, Tyr199^	Rabbit	1:500	PA5-118,549	Thermo/Invitrogen
Anti-AKT	Mouse	1:1000	60,203–2-Ig	Proteintech
Anti-p-AKT^Ser473^	Mouse	1:1000	66,444–1-Ig	Proteintech
Anti-NF-κB p65	Rabbit	1:1000	10,745–1-AP	Proteintech
Anti-TNF-α	Mouse	1:1000	60,291–1-Ig	Proteintech
Anti-E-cadherin	Rabbit	1:1000	PA5-32,178	Thermo/Invitrogen
Anti-N-cadherin	Mouse	1:1000	MA1-91,128	Thermo/Invitrogen
Anti-TGF-β	Rabbit	1:1000	MA5-15,065	Thermo/Invitrogen
Anti-Twist	Mouse	1:500	MA5-17,195	Thermo/Invitrogen
Anti-Snail	Rabbit	1:500	MA5-14,801	Thermo/Invitrogen
Anti-CD44	Rabbit	1:1000	15,675–1-AP	Proteintech
Anti-CD133	Rabbit	1:1000	18,470–1-AP	Proteintech
Anti-Nanog	Rabbit	1:500	14,295–1-AP	Proteintech
Anti-β-actin	Mouse	1:2500	MA5-15,739	Thermo/Invitrogen
Anti-Mouse IgG	Goat	1:5000	31,430	Thermo/Invitrogen
Anti-Rabbit IgG	Goat	1:5000	31,460	Thermo/Invitrogen

^*^Antibodies used for immunoblotting were from Novus (St.Louis/USA), Thermo/Invitrogen (Carlsbad/USA) and Proteintech (Chicago/USA)

### Bioinformatic analysis

CER’s canonical SMILES structure was obtained from PubChem (https://pubchem.ncbi.nlm.nih.gov/) and searched in SwissTargetPrediction (http://www.swisstargetprediction.ch/; screened based on probability > 0) to predict target proteins. Glioma-associated genes were identified using GeneCard (https://www.genecards.org). Common targets from CER and glioma were found using Venn diagrams (http://bioinformatics.psb.ugent.be/webtools/Venn/). Protein–protein interaction networks were analyzed with STRING software v12.0 (http://string-db.org). Gene ontology (GO; a standardized system for classifying gene functions into biological processes, molecular functions, and cellular components) and Kyoto Encyclopedia of Genes and Genomes (KEGG) were utilized for pathway enrichment analyses and performed using ShinyGO v0.8 (http://bioinformatics.sdstate.edu/go/). Each category was ranked by significance, and the top 10 items were displayed in charts.

### Statistical analysis

Quantitative data were presented as mean ± SD from at least three independent experiments. The IC_20_ and IC_50_ values were determined through nonlinear regression analysis of the sigmoidal dose–response curve. Statistical evaluation was done with one- or two-way ANOVA followed by Tukey post hoc-test using GraphPad Prism v10.2. *P* < 0.05 was considered as statistically significant.

## Results

### Cerulenin treatments decrease the viability of glioma cells in a dose- and time-dependent manner

**Fig. 1:**
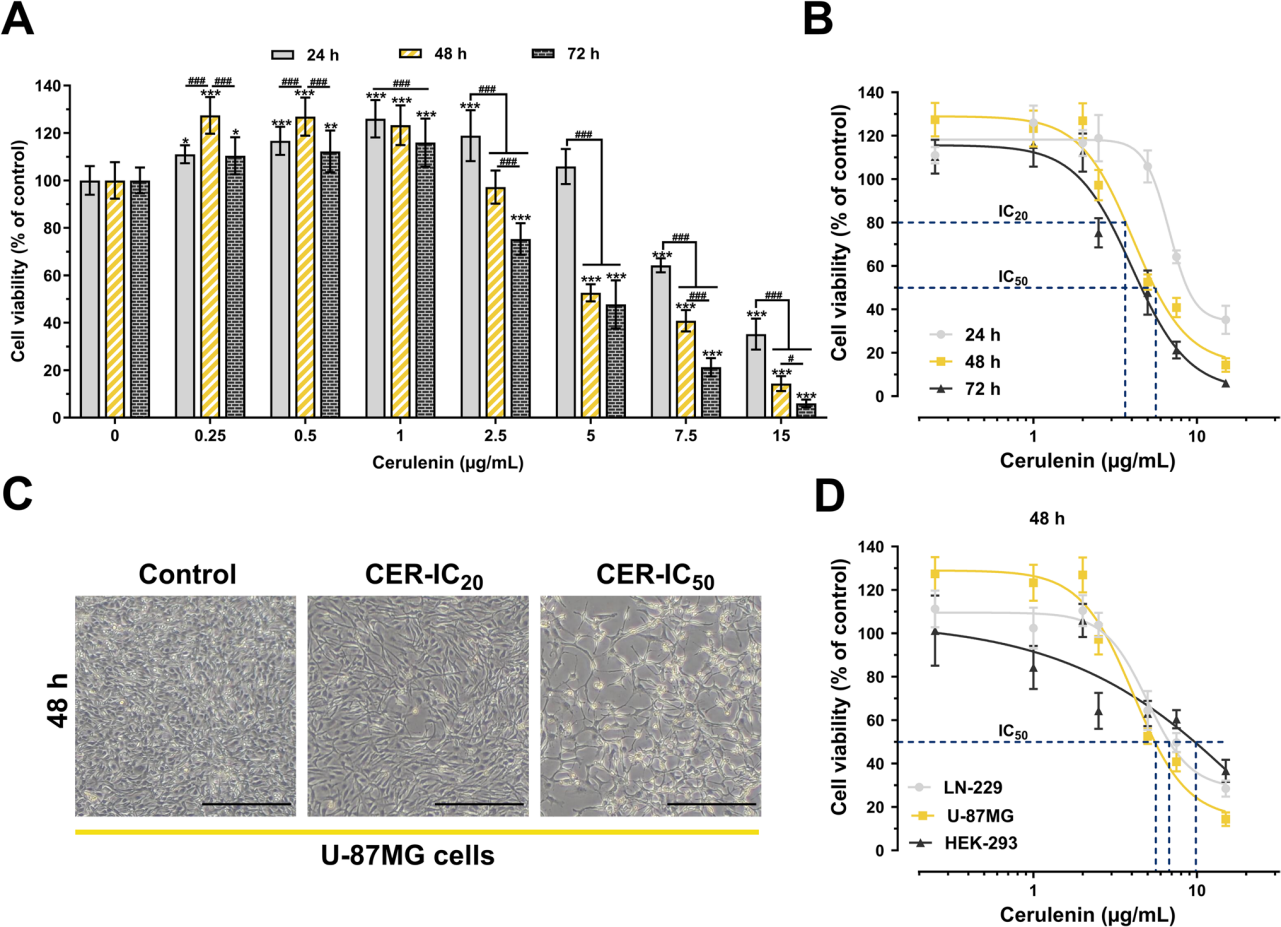
Cerulenin (CER) reduces the viability of U-87MG glioma cells in a dose- and time-dependent manner. **A** U-87MG cells were treated with increasing concentrations (0–15 μg/mL) of CER for 24 h, 48 h, and 72 h, and an MTT assay was performed to detect cell viability **B** Dose–response curves for detecting IC_20_ and IC_50_ values of CER treatments. **C** After treatments of selected CER doses (IC_20_ = 3.6 μg/mL and IC_50_ = 5.55 μg/mL) for 48 h, the cell morphological alterations were observed under an inverted light microscope (× 40, scale bar: 400 µm). DMSO (D, 0.06%; applied as the vehicle in the control group) had no effect on cell morphology and viability. **D** Dose–response curves of CER treatments for 48 h in human glioma cell lines U-87MG and LN229, as well as the normal human cell line HEK-293. Data are presented as mean ± SD (*n* = 10). ***P < 0.001, **P < 0.01, *P < 0.05, versus control. ^###^*P* < 0.001, ^#^*P* < 0.05 versus treatment time. Statistical evaluation was performed with two-way ANOVA (Tukey post hoc-test)

The MTT assay showed the time- and dose-dependent cytotoxic effects of CER on U-87MG cells (Fig. 1A). CER reduced glioma viability starting at 7.5 µg/mL for 24 h treatment, while effects were detected at lower doses for 48 h and 72 h therapy periods. Due to the similar sigmoidal dose–response curves of CER at 48 h and 72 h, further experiments used low (IC_20_ value) and high (IC_50_ value) doses for a 48 h treatment (Fig. 1B, Table 2). The maximum DMSO concentration in the culture medium was 0.06% and did not affect cell viability. Besides, control cells (treated with %0.06 DMSO) exhibited normal morphology (well spread and flattened), whereas changes in cells’ shape and adhesion capacity were observed in groups treated with 3.6 µg/mL and 5.5 µg/mL of CER. The highest impact was seen at the IC_50_ dose with more cell shrinkage and cellular irregularity (Fig. 1C). Besides, after 48 h of CER treatment, the IC_50_ values for LN-229 (a different GB cell line from U-87MG) and normal human HEK-293 cells (which exhibit an epithelial morphology similar to that of GB cells) were 6.8 µg/mL and 9.7 µg/mL, respectively. Among these three cell lines, U-87MG demonstrated the lowest IC_50_ value (Fig. 1D).

**Table 2:** IC_20_ and IC_50_ values (mean ± SD) of Cerulenin treatments in U-87MG human glioblastoma cells

	IC_20_ value (µg/mL)	µM	IC_50_ value (µg/mL)	µM
24 h	6.55 ± 0.22	= 29.34	8.37 ± 0.74	= 37.49
48 h	3.6 ± 0.34	= 16.12	5.55 ± 0.25	= 24.86
72 h	3.19 ± 0.19	= 14.29	4.74 ± 0.29	= 21.23

### Cerulenin treatments inhibit fatty acid synthesis in dose-dependent manner

**Fig. 2:**
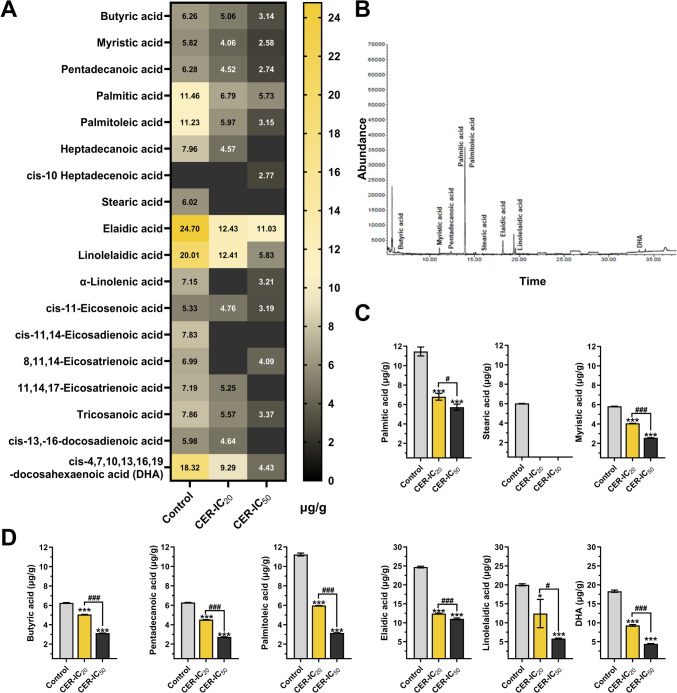
Cerulenin (CER) dose-dependently inhibits fatty acid synthesis in U-87MG glioma cells. **A** Fatty acid profile (in the heatmap chart) by detecting with GC–MS analysis. **B** GC–MS chromatogram of fatty acids in lipid samples from U-87MG cell pellet. Peaks of palmitic acid and palmitoleic acid from lipid samples of control cells are displayed at RT 13.48 and 14.6 min, respectively. **C** Quantitative analysis of the palmitic acid, stearic acid, and myristic acid, which are the main products during the catalysis of fatty acid synthase (FASN). **D** Quantitative analysis of the butyric acid, pentadecanoic acid, palmitoleic acid, elaidic acid, linolelaidic acid, and DHA. Data are presented as mean ± SD (*n* = 3). ***P < 0.001, *P < 0.05, versus control (represents vehicle control). ^###^*P* < 0.001, ^#^*P* < 0.05, indicate comparison between CER doses. Statistical evaluation was performed with one-way ANOVA (Tukey post hoc-test)

Changes in the fatty acid content of U-87MG cells after CER treatments were detected by GC–MS analysis and results presented in the heatmap chart (Fig. 2A). The levels of palmitic acid (C16:0), stearic acid (C18:0), and myristic acid (C14:0), which are main products during the catalysis of FASN[4], were markedly lower in both CER-IC_20_ and CER-IC_50_ groups compared to control (*P* < 0.001); showing evidence that CER inhibits FASN (Fig. 2B). Palmitic acid and myristic acid levels decreased by ~ 50%, while stearic acid was completely depleted. As shown in Fig. 2C, other fatty acids, including butyric acid, pentadecanoic acid, and palmitoleic acid, were significantly reduced due to CER treatments (*P* < 0.001). Some were not even detected in CER-IC_20_ and/or CER-IC_50_ groups. Remarkably, the levels of elaidic acid, linolelaidic acid, and DHA, which were higher than other fatty acids’ amounts in the U-87MG cells, decreased dramatically after CER treatments (*P* < 0.001). Additionally, some fatty acids in the standard (caproic acid, caprylic acid, capric acid, undecanoic acid, lauric acid, tridecanoic acid, myristoleic acid, cis-10 pentadecenoic acid, oleic acid, linoleic acid, arachidic acid + γ-linolenic acid, heneicosanoic acid, behenic acid, erucic acid + cis-5,8,11,14-eicosatetraenoic acid, cis-5,8,11,14,17-eicosapentaenoic acid/EPA, lignoseric acid, and nervonic acid) were not detected in any group.

### Cerulenin treatments decrease colony formation, migratory, and differentiation characteristics of human glioblastoma cells

**Fig. 3:**
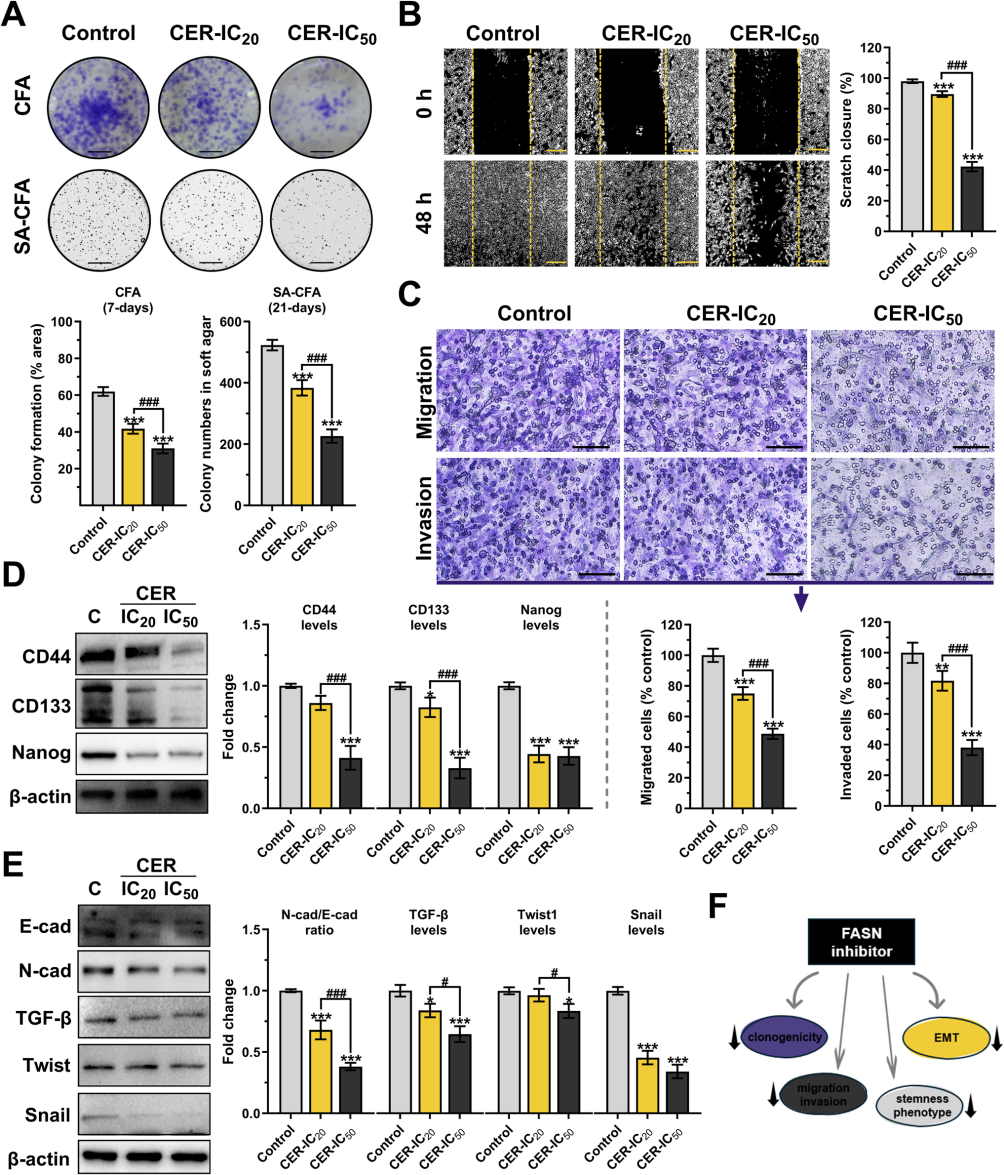
Cerulenin (CER) decreases colony formation, migratory, and differentiation characteristics of U-87MG glioma cells. **A** The colony-forming ability of the cells was evaluated with CFA in 2D-culture (crystal violet staining on day 7; *n* = 5) and SA-CFA in soft agar (3D-culture, nitrotetrazolium blue chloride staining on day 21, *n* = 5; scale bar: 800 µm). **B** The migratory ability of the cells was evaluated by scratch-wound assay (*n* = 5; × 40, scale bar: 400 µm). **C** Transwell migration and invasion assay (*n* = 5; × 200, scale bar: 200 µm). **D** Western blot analysis (*n* = 3) of CSC markers (CD44, CD133, and Nanog). **E** Western blot analysis (*n* = 3) of EMT markers (N-cadherin/E-cadherin, Snail, Twist1, TGF-β). β-actin was used as a loading control. **F** Shape showing decreased clonogenicity, migration, EMT, and CSC status upon FASN inhibition. Data are presented mean ± SD. ***P < 0.001, *P < 0.05, versus control (C; represents vehicle control). ^###^*P* < 0.001, ^#^*P* < 0.05, indicate comparison between CER doses. Statistical evaluation was performed with one-way ANOVA (Tukey post hoc-test). *CFA C*olony formation assay, *CSC *Cancer stem cell, *EMT *Epithelial-mesenchymal transition, *FASN *Fatty acid synthase, *SA-CFA *Soft agar CFA

After CER treatments, the colony-forming abilities of U-87MG cells were tested in 2D- and 3D-cultures (Fig. 3A). At the end of the 7th day, the control cells in the 2D-culture covered 61.98% area, while the cells treated with CER (IC_20_ and IC_50_ doses) covered 41.72% and 31% area (*P* < 0.001), respectively. The ability to form colonies on soft agar (in 3D-culture for 3 weeks) was evaluated to determine whether applied CER treatment affected anchorage-independent growth. The least number of colony formation was observed in the CER-IC_50_ group (226.2 ± 22.03 colonies, *P* < 0.001). As shown in Fig. 3B, the scratch migration assay revealed that CER therapy markedly reduced the scratch closure rate in dose-dependent manner in glioma cells. Compared to control, scratch closure rates were 89.64% and 42.3% in the CER-IC_20_ and CER-IC_50_ groups. The transwell chamber assay showed CER dose-dependently reduced gliomas’ migrative and invasive ability; migrated and invaded cells in the CER-IC_50_ group were 48.76% and 38.12% of control, respectively (Fig. 3C). Besides, the differentiation characteristics of untreated and treated glioma cells were evaluated by detecting the marker protein levels of stemness (Fig. 3D) and EMT (Fig. 3E), which are influential in glioma aggressiveness and spread[18]. The cancer stem cell (CSC) marker levels decreased after CER treatments. While the effect of the IC_20_ dose on CD44 and CD133 levels was negligible, a similar impact on Nanog levels was detected in the CER-IC_20_ and CER-IC_50_ groups. Notably, CD44 (2.45-fold, *P* < 0.001), CD133 (3.03-fold, *P* < 0.001), and Nanog (2.32-fold, *P* < 0.001) levels were markedly reduced in treated glioma cells with an IC_50_-value of CER compared to the control. Furthermore, CER significantly decreased N-cadherin (a mesenchymal marker)/E-cadherin (an epithelial marker) ratio (2.63-fold, *P* < 0.001), TGF-β (1.56-fold, *P* < 0.001), Twist1 (1.19-fold, *P* < 0.05), and Snail (2.94-fold, *P* < 0.001) levels in dose-dependent manner. As briefly stated in Fig. 3F, the FASN inhibitor CER markedly suppressed the differentiation characteristics of glioma cells by reducing EMT- and stemness-related mesenchymal marker levels, thus attenuating the cells’ colony-forming capacity, and migrative and invasive ability.

### Cerulenin treatments induce apoptosis and autophagy in human glioblastoma cells

**Fig. 4:**
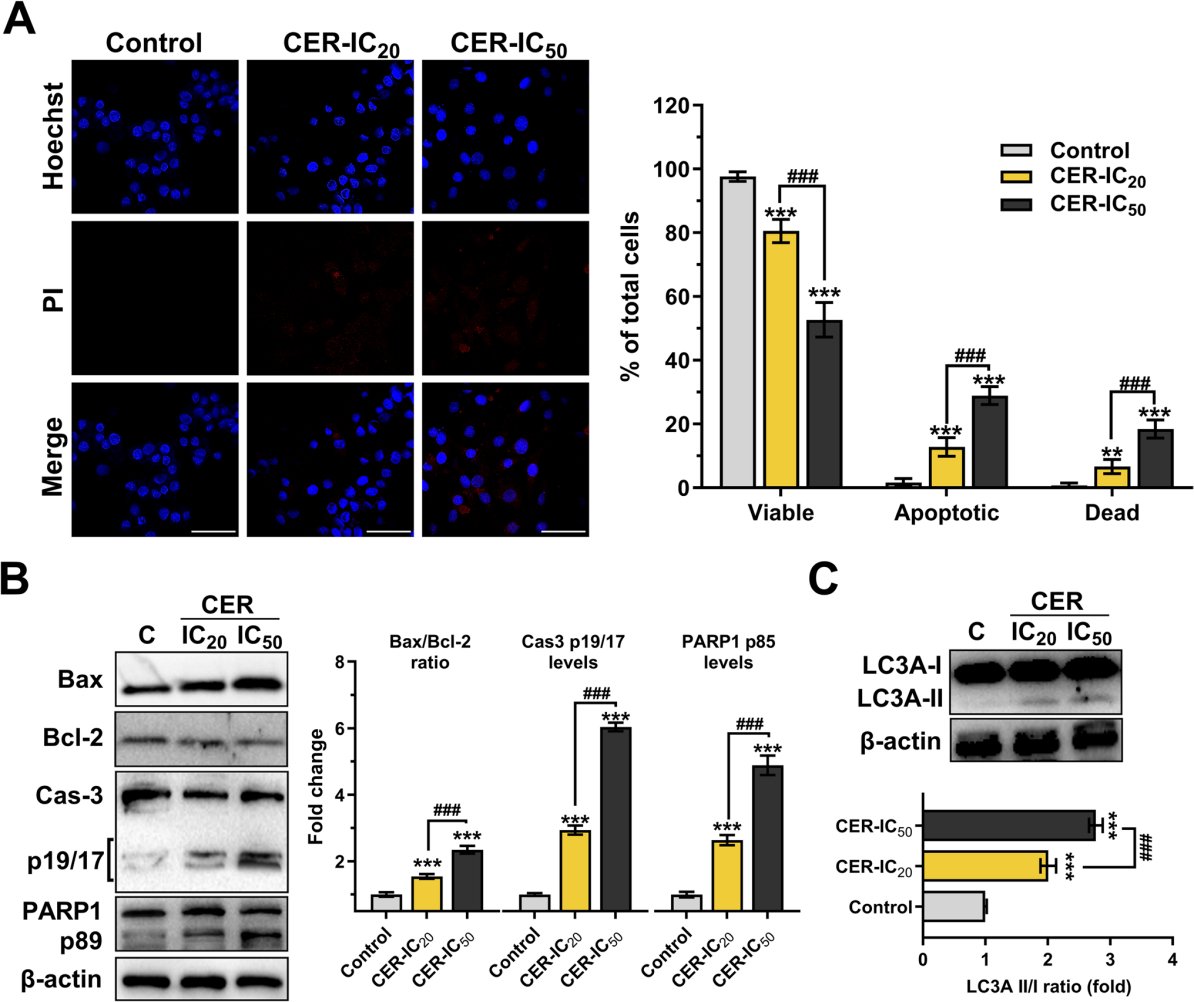
Cerulenin (CER) induces apoptosis and autophagy in U-87MG glioma cells. **A** Double staining of the cells with Hoechst (blue-fluorescent dye) and PI (red-fluorescent dye) for determining apoptosis and quantitative analysis (*n* = 5; × 400, scale bar: 50 µm). **B** Western blot analysis of Bax/Bcl-2 ratio, and caspase-3 and PARP-1 fragments. **C** Western blot analysis (*n* = 3) of LC3A-II/I ratio to evaluate autophagy. β-actin was used as a loading control. Data are presented mean ± SD. ***P < 0.001, **P < 0.01, versus control (C; represents vehicle control). ^###^*P* < 0.001, indicate comparison between CER doses. Statistical evaluation was performed with one-/two-way ANOVA (Tukey post hoc-test)

As shown in Fig. 4A, Hoechst staining revealed increased apoptotic bodies and nuclear condensations, while PI staining showed dead cells in CER-treated cells. While 12.8% (*P* < 0.001) and 28.92% (*P* < 0.001) of apoptotic cells were detected in the CER-IC_20_ and CER-IC_50_ groups, respectively, dead cells were 6.68% (*P* < 0.01) and 18.42% (*P* < 0.001) of total cells, respectively. The levels of important marker proteins in the apoptotic pathway were determined by immunoblotting (Fig. 4B). As expected, the CER-IC_50_ treatment induced apoptosis more than CER-IC_20_; it caused a 2.35-fold increase in Bax/Bcl-2 ratio (*P* < 0.001) and increased active fragments of caspase-3 (6.04-fold, *P* < 0.001) and PARP1 (4.88-fold, *P* < 0.001). To detect autophagic activity after CER treatment, the soluble form (LC3A-I) and the autophagosome membrane-bound form (LC3A-II) of LC3A were analyzed by western blotting. The conversion from LC3-I to LC3-II in treated cells (CER-IC20 and CER-IC50 groups) were 2.01- and 2.77-fold increases compared to control cells (*P* < 0.001), respectively (Fig. 4C).

### Cerulenin treatments induce cellular stress response in human glioblastoma cells

**Fig. 5:**
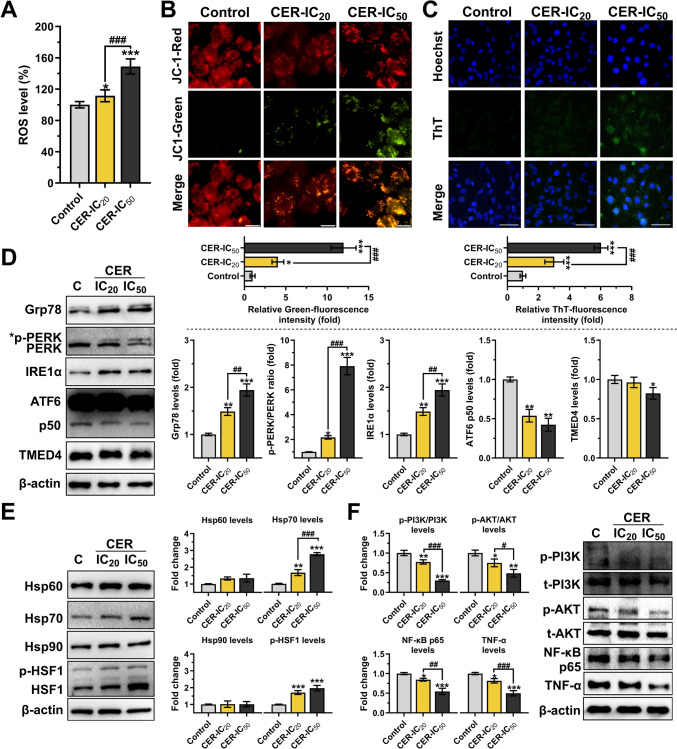
Cerulenin (CER) reduces PI3K/AKT signaling, induced ER stress, and increase stress protein levels in U-87MG glioma cells. **A** The intracellular ROS levels were determined by kinetic measurements of DCF fluorescence intensity (*n* = 6). **B** The mitochondrial membrane potential (MtMP) was evaluated with JC-1 staining (*n* = 3; × 1000, scale bar: 10 μm). **C** The misfolded protein aggregates were evaluated with ThT staining (*n* = 5; × 400, scale bar: 50 μm). **D** Western blot analysis (*n* = 3) of ER stress markers (Grp78, p-PERK, IRE1α, cleaved-ATF6, and TMED4). **E** Western blot analysis (*n* = 3) of stress proteins (Hsp60, Hsp70, Hsp90, and p-HSF1). **F** Western blot analysis (*n* = 3) of p-PI3K, p-AKT, NF-κB p65, and TNF-α. β-actin was used as a loading control. Data are presented mean ± SD. ***P < 0.001, **P < 0.01, *P < 0.05, versus control (C; represents vehicle control). ^###^*P* < 0.001, ^##^*P* < 0.01, ^#^*P* < 0.05, indicate comparison between CER doses. Statistical evaluation was performed with one-way ANOVA (Tukey post hoc-test)

To evaluate cellular stress response after CER treatments, ROS levels, MtMP status, ER stress, and target protein expression profiles were analyzed. As shown in Fig. 5A, CER treatments markedly increased ROS generation relative to basal control levels (for CER-IC20, 111.47%, *P* < 0.05; for CER-IC50, 148.95%, *P* < 0.001). Additionally, the effect of CER on MtMP was analyzed by JC-1 staining (Fig. 5B; the red-to-green fluorescence intensity depends on membrane integrity). Treated cells with CER-IC_20_ and CER-IC_50_ showed a loss of MtMP in a dose-dependent manner with fold changes of 4.05 (*P* < 0.05) and 11.98 (*P* < 0.001), respectively. As indicated in Fig. 5C (ThT staining), the intensity of ThT fluorescence after CER-IC_20_ and CER-IC_50_ treatments increased by 3.02- (*P* < 0.001) and 6.03-fold (*P* < 0.001), respectively, compared to the control; showing that increased misfolded protein aggregates reflecting the onset of ER stress[23]. Immunoblotting analysis (Fig. 5D) confirmed CER-induced ER stress at the molecular level. Typical markers of ER stress, Grp78, PERK, IRE1α, ATF6, and TMED4, were evaluated for UPR signaling. Notably, CER-IC_50_ treatment significantly increased p-PERK (7.91-fold, *P* < 0.01), IRE1α (1.95-fold, *P* < 0.001), and Grp78 (1.93-fold, *P* < 0.05), but decreased cleaved-ATF6 (2.36-fold *P* < 0.01) and TMED4 (1.20-fold, *P* < 0.05) levels. Besides, as shown in Fig. 5E, CER treatments caused the increase in the levels of Hsp70 (for CER-IC_50_, 2–76-fold, *P* < 0.001) and p-HSF1 (for CER-IC_50_, 1–97-fold, *P* < 0.001), which role in response to cellular stress[14], but Hsp60 and Hsp90 levels did not change. Moreover, Fig. 5F showed the downregulation of the PI3K/AKT pathway (for CER-IC50, 3.30-fold and 2.06-fold, respectively, *P* < 0.001), a signaling pathway whose expression changes in response to many stress signals [24]. A decrease in NF-κB p65 (1.85-fold, *P* < 0.001) and TNF-α (2.04-fold, *P* < 0.001) levels was also observed due to the CER-IC_50_ treatment.

### Cerulenin and glioma-related targets and their functional classifications

**Fig. 6:**
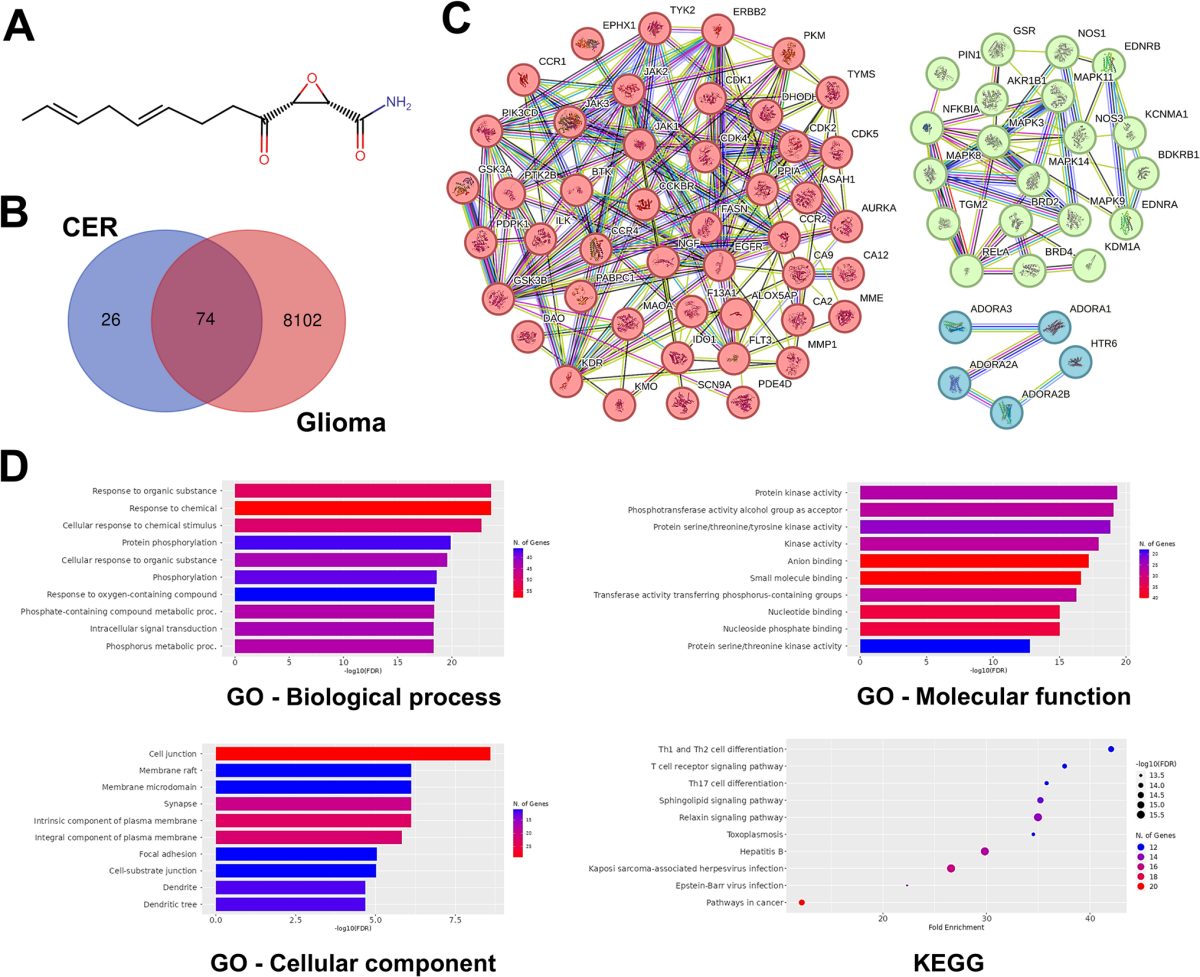
Network pharmacology analysis for predicting the possible intersection of target genes and biological pathways related to cerulenin (CER) and glioma. **A** The molecular structure of CER. **B** Venn diagram identifying the intersection of CER and glioma-related target genes **C** The protein–protein interaction (PPI) network detected by STRING software using intersecting target genes. **D** The result of GO (the terms are biological process, cellular components, and molecular function) and KEGG pathway enrichment analyses. *GO *Gene ontology, *KEGG *Kyoto Encyclopedia of Genes and Genomes

First, the potential targets of CER and glioma-related targets were detected and then analyzed to identify common candidates; the 74 protein targets were identified (Fig. 6A, B). As shown in Fig. 6C, the most notable among them were FASN, cell division cycle proteins (CDK1, CDK2, CDK4, CDK5), cell membrane receptors (EGFR, ErbB2), and cell signaling molecules (MAPKs, PI3K, and NF-κB p65). These potential targets and their associated functions, biological processes, and cellular components were further investigated through GO and KEGG pathway enrichment analyses (Fig. 6D). Remarkably, protein kinase activity, protein phosphorylation, cell junctions, and cell differentiation mechanisms came to the fore. Bioinformatic analysis showed that these targets and pathways may play a role in the mechanism of action of cerulenin against glioma progression and deserve further investigation.

## Discussion

One area of interest in cancer research is targeting the metabolism of tumor cells. Focusing on the metabolic pathways, including glycolysis and fatty acid synthesis, of fast-growing glioblastoma cells, which have a higher demand for biomolecules than healthy cells, is a promising therapeutic strategy [2]. Cerulenin is a natural antimycotic found in the fungus *Cephalosporium caerulens* and an inhibitor that targets FASN by binding covalently to the cysteine thiol of the active site. CER is selectively toxic to cancer cells in vitro. FASN is expressed at low levels in most healthy tissues, and the diet supplies most of the required lipids. Thus, endogenous fatty acid biosynthesis is downregulated. In contrast, FASN expression becomes overexpressed in cancer cells due to rapid growth and increased metabolic burden. The expression level of FASN also increases in the early stages of cancer and is associated with poor prognosis, indicating that targeting FASN can be a crucial therapeutic option for aggressive GB cells by providing a selective advantage [2, 7].

Herein, CER treatments decreased the viability of U-87MG human glioma cells in a dose- and time-dependent manner. The sigmoidal dose–response curve revealed a low dose (IC_20_ value) and a high dose (IC_50_ value) for further experiments with 48 h of CER treatment (Fig. 1A–C). These experiments were conducted using U-87MG cells with a p53-wild type/PTEN-mutant profile, which is common in glioblastoma patients [25, 26]. U-87MG was also selected for this study due to its extensive research and sequenced genome [27]. Besides, MTT analysis revealed that, CER treatments resulted in higher IC_50_ values for LN-229 cells (a human GB cell line; p53-mt/PTEN-wt) and HEK-293 cells (a normal human cell line which exhibit an epithelial morphology similar to GB cells [28, 29] compared to U-87MG cells (p53-wt/PTEN-mt) (Fig. 1D). As shown in our previous report, the difference in p53 status between U-87MG and LN-229 GB cells [30] may be a reason for the difference between the inhibitory doses. As a tumor suppressor, p53 expression was shown to increase in p53 wild-type cells during FASN inhibition, thus contributing to the stoppage of cancer’s progression. Apart from the p53-p21 pathway, modulation of ERK1/2 and NF-κB signaling pathways was also shown to be involved in this antitumoral impact [31]. Moreover, CER was demonstrated to induce varying levels of apoptosis in different wild-type p53 and mutant p53 tumor cell lines, while normal human keratinocytes and fibroblasts exhibit greater resistance to this apoptotic effect [32]. One reason healthy cells are less impacted by FASN inhibition might be their lower metabolic load than cancer cells [33]. Another reason could be that leptin hormone receptors, which control food metabolism, are highly expressed in cancer cells compared to healthy cells, and CER mimics leptin to generate a satiety signal in these cells, potentially leading to reduced nutrient intake and subsequently impaired metabolic activity [34, 35].

GS-MS analysis shown in Fig. 2 confirmed the FASN inhibitory function of CER with a decreased fatty acid profile of the treated cells. Supporting documents showed that FASN inhibition by CER slows proliferation of various cancer cells, including breast [36], colorectal [37], glioma [8], liver [38], and melanoma [39]. Reducing the proliferation and motility of aggressively growing GB cells is critical for therapy efficacy. Clonogenecity is a characteristic of cancer and cancer stem cells that display both anchorage-independent and dependent proliferation and differentiation. Additionally, mesenchymal GSCs (glioma stem cells) are enriched in genes associated with these properties [1]. In this regard, monitoring change markers of differentiation characteristics, as well as increasing invasion and migration is one of the crucial information providers about the prognosis of the disease. Notably, EMT, providing mesenchymal properties from epithelial cells, is a phenotypic conversion that accelerates tumor invasion and metastasis, and also its activation is associated with generating CSCs [40]. Herein, CER treatments reduced both 2D- and 3D-colony formation, migration and invasion of U-87MG cells (Fig. 3A–C). By supporting us, the FASN inhibitor CER was reported to decelerate 2D-colony formation in glioma [6] and breast [36] cancer cells. In this study, we also showed that CER treatment caused a decrease in the levels of mesenchymal (N-cadherin/E-cadherin ratio, Snail, Twist1, and TGF-β) and stemness (CD44, CD133, and Nanog) markers in U-87MG cells (Fig. 3D, E). Our data was supported by similar findings regarding EMT and CSC markers in breast cancer [17] and glioma [8]. This evidence shows that FASN inhibition can attenuate CSC function by reducing clonogenicity, migration, and EMT markers in aggressive glioblastoma cells.

The primary goal in treating cancer cells is to induce apoptosis [1]. Fluorescence microscopy results showed a significantly higher number of apoptotic cells in CER-treated cells, with nuclear condensation and fragmentation as markers of apoptosis by Hoechst staining (Fig. 4A) as an expected effect [3]. Immunoblotting results also revealed a significant presence of apoptosis with increased levels of apoptotic markers (Fig. 4B). Notably, BCL-2 family proteins strictly control mitochondrial apoptosis through a network of protein interactions [41]. CER treatment increased the Bax/Blc-2 ratio (which indicates mitochondrial damage) and cleaved-caspase-3 and PARP1 fragments, thus inducing intrinsic apoptosis. Supporting studies showed that treating glioma cells with CER triggered S-phase cell arrest and apoptosis [6] and resulted in G2-phase cell arrest and apoptosis [11]. Furthermore, autophagy was also increased based on immunoblotting data (Fig. 4C), which showed increased LC3A-II cleaved form (an essential step in autophagosome formation). Autophagy is a dynamic process that plays a role in cancer cells’ survival and death. It is linked to ER stress, intracellular ROS, and apoptosis [42]. Moreover, high levels of mitochondrial ROS can activate apoptosis and autophagy pathways to trigger cell death [43]. Our results showed that increased ROS and UPR response due to inhibition of the lipid metabolism (Fig. 5) may be involved in the mechanism of cell death induced in glioma cells.

It is known that there is a strong positive correlation between MtMP and ROS production [43]. Previous studies revealed that FASN inhibition causes MtMP disruption and ROS burst in cancer cells, including breast cancer [10], melanoma [44], and neuroblastoma [32]. Our study showed that, for the first time, CER disrupted MtMP and triggered ROS burst in human glioma cells (Fig. 5A, B), consistent with the above literature. Moreover, ER stress also causes mitochondrial dysfunction and increases ROS production [42]. Our findings revealed that CER treatments increased accumulation of unfolded/misfolded proteins (Fig. 5C) that cause UPR [23]. Ultimately, increased Grp78 (glucose-regulated protein 78), p-PERK (protein kinase RNA-like ER kinase), and IRE1α (inositol-requiring enzyme type-1α) levels were observed, while ATF6 p50 (activating transcription factor 6) and TMED4 (transmembrane emp24 domain-containing protein) levels were reduced (Fig. 5D). As known, ER stress is an important response for cancer cells and is associated with both tumor growth and death. Master regulator Grp78 and its copartners (especially PERK, IRE1a, and ATF6; ER transmembrane proteins) respond to ER stress to activate lipid synthesis, autophagy, redox homeostasis, or apoptosis. Indeed, ER stress can trigger cell death when an unaffordable burden is formed [23]. Walter et al. [45] reported that ATF6 silencing resulted in uncontrolled IRE1-reporter activity, increased X box-binding protein 1 (XBP1) splicing and apoptosis, which coincides with our findings. Chen et al. [11] also revealed that CER increased cell arrest and apoptosis through enhanced ER stress (with increased IRE1, XBP1 and CHOP levels). Furthermore, TMED4 is induced by UPR stressors and serves as cargo proteins by shipping proteins between the ER and Golgi apparatus [46]. Decreased TMED4 due to CER treatment may have contributed to slowing glioma progression, as supported by Ullah et al.’’ [47] finding that TMED4 expression is negatively correlated with the survival rate of GB patients.

Heat shock/stress proteins (Hsp) are closely associated with UPR and ER stress [13], treatment resistance, motility, and stem cell behavior [14]. However, the information on FASN inhibition on Hsp expression is insufficient. Only two studies reported that CER increases Hsp70 levels in prostate cancer cells [15] and chicken muscle [16]. For the first time, this study revealed that Hsp70 and p-HSF1 (heat shock transcription factor 1) levels increased in glioma cells due to CER-mediated FASN inhibition, while no significant change was observed in Hsp60 and Hsp90 levels (Fig. 5E). It was unsurprising that in addition to the increased ER chaperone Grp78 (an Hsp70 family member) due to ER stress [23], Hsp70 levels and the phosphorylated active form of the transcription factor HSF1 increased. The levels of these stress proteins may have increased to alleviate the burden of the aggregates (Fig. 5C) formed by the rising unfolded/misfolded proteins in cells [48]. However, the net result was reduced proliferation and increased cell death (Fig. 3, 4). Moreover, the unchanged levels of Hsp90, which effectively keeps HSF1 inactive in the cytoplasm, may have served to release HSF1 and switch to its active form by trimerization and phosphorylation [49]. Additionally, UPR occurs not only in the ER but also in mitochondria. The lack of change in the levels of Hsp60, which has a crucial function in folding proteins in mitochondria, may not have been enough to overcome the mitochondrial stress, thus causing increased ROS and MtMP damage and triggering apoptosis [50]. These findings deserve further research to elucidate the intracellular effects of these changes in Hsp levels.

The PI3K/AKT pathway in cancer cells is an over-activated intracellular pathway contributing to carcinogenesis, proliferation, invasion, and metastasis of tumor cells. Its downstream regulator, NF-κB provides cancer cells a survival advantage by upregulating antiapoptotic genes. This axis also contributes to the inflammatory response by affecting the secretion of proinflammatory cytokines such as TNF-α [24]. In our study, for the first time, the FASN inhibitor CER reduced the levels of p-PI3K (a lipid kinase family member), p-AKT, NF-κB p65 active subunit, and TNF-α in human glioma cells (Fig. 5F). Supportively, FAS inhibition was reported to suppress osteosarcoma cell invasion and migration via downregulation of the PI3K/Akt signaling pathway [51]. Besides, since PI levels in glioblastoma tumors are higher than in healthy brain tissue [52], stopping FASN-mediated PI3K signaling increases the therapeutic importance of CER. Furthermore, it is well-known that ER stress suppresses the PI3K/AKT signaling pathway, leading to the induction of autophagy and apoptosis in cancer cells [53]; our results support this. Herein, we provided evidence that CER increased protein aggregates, triggered UPR in both ER and mitochondria, led to MtMP disruption and ROS burst, and downregulated PI3K/Akt/NF-κB pathway. Our network pharmacological analysis suggested PI3K and NF-κB p65 as potential targets of CER in glioma (Fig. 6), consistent with the data we obtained (Fig. 5). The network analysis also identified significant some proteins, including CDKs [54], EGFR [55], and MAPK [31], which were consistent with previous studies and could be considered for further research. Moreover, KEGG database analysis revealed the apparent involvement of CER in the immune system and signaling pathways in cancer cells. Supportively, a previous study demonstrated that CER influences the immune system by impacting T helper cells [56]. Our wet-lab results indicate that the relationship between CER and signaling pathways, ER stress, CSC, and HSPs (which are linked to cancer progression) is consistent with the findings of KEGG analysis.

The presented study has some limitations. Although a few in vivo studies demonstrated CER’s potential effects [51, 57], chemical instability makes it unsuitable as a systemic therapeutic agent [58]. Besides, CER exhibits a toxic effect on healthy cells, but its pronounced efficacy and higher toxicity in cancer cells underscore its potential as an anti-cancer agent [32]. Therefore, using carrier systems such as nanotechnological formulations [59] or using derivatives such as C75 [58] will increase bioavailability. Previous studies on CER effects in various cancer types, including gliomas, have typically used CER doses of 2–20 µg/mL in vitro experiments [8, 36–39]; the doses applied in this study fall within this range. Additionally, doses of 2–10 µg/mL of CER were employed in sphere culture experiments [8, 11], while doses of 15–40 mg/kg were administered for in vivo studies [37, 51]. Although our data are limited to in vitro administration of low and high doses of CER, the results on the molecular action mechanism direct further research to increase the availability of cerulenin for treating GB. In this context, further studies on different doses may be needed to analyze the molecular effects of CER further 3D in vitro and in vivo studies. Another limitation of our study is that CER does not demonstrate the effect of drug resistance mechanisms in cancer therapy. Some previous studies on CER impacts with cancer drugs, such as trastuzumab/breast cancer [31], 5-FU/breast cancer [60], and temozolomide + radiotherapy/glioblastoma [11], showed increased drug effectivity; these studies commonly found changes in signaling pathways and ER stress with FASN inhibition. Another critical research study showed that FASN inhibition reduces glioma stem cells responsible for treatment resistance, migration, and invasiveness [8]]. Our results show that CER treatments caused changes in signaling pathways and increased ER stress. Besides, our findings regarding the expression changes in EMT [61], CSC markers [62], and HSPs [63], which have a crucial role in drug resistance, suggest that CER may contribute to reducing drug resistance.

Consequently, the present results showed that CER inhibited FASN, slowed glioma proliferation, decreased migration ability, and formed 2D and 3D colonies. The expression of differentiation markers (EMT and CSC) decreased, thus reducing the aggressiveness of glioma. Moreover, CER treatments caused downregulation of the PI3K/AKT/NF-κB axis (one of the targets offered by network pharmacology) and induced apoptosis and autophagy via MtMP disruption, ROS burst, protein aggregation (UPR), and ER stress. Despite increased Hsp70 and p-HSF1 levels, the blocking fatty acid synthesis promoted cell death. The results of studies targeting a specific fatty acid also agreed with our findings. Huang et al. [64] reported that palmitic acid levels in breast cancer cells were negatively correlated with ER stress and apoptosis. The lipid regulator butyric acid was shown to reduce ER stress in cells [65]. Besides, a decrease in the levels of main products of FASN, palmitic acid, stearic acid, and myristic acid, induced apoptosis in mouse embryonic fibroblasts by triggering p21 and Bax expressions, while treatments with the fatty acids reduced the apoptotic protein levels in breast cancer cells [66]. All these prove how cancer cell progression weakens without a or more fatty acids, highlighting the therapeutic effectiveness of stopping multitarget synthesis using CER (focusing on the metabolic activity) in treating GB. Our findings suggest that CER is worth further investigation due to its potential to be used both alone and in combination with other drugs.

## Data Availability

The presented data is available upon request from the corresponding authors.

## Abbreviations

AKT/PKB: Protein kinase B
CER: Cerulenin
CFA: Colony formation assay
CSC: Cancer stem cell
EMT: Epithelial-mesenchymal transition
ER: Endoplasmic reticulum
FA: Fatty acids
FASN: Fatty acid synthase
GB: Glioblastoma
Hsp: Heat shock protein
IC_50_: Half maximal inhibitory concentration
NF-κB: Nuclear factor-kappa B
PI3K: Phosphatidylinositol 3-kinase
ROS: Reactive oxygen species
SA-CFA: Soft agar colony formation assay
TMED4: Transmembrane emP24 trafficking protein 4
UPR: Unfolded protein response
